# Phosphorylation of eIF2α attenuates statin-induced apoptosis by inhibiting the stabilization and translocation of p53 to the mitochondria

**DOI:** 10.3892/ijo.2013.1792

**Published:** 2013-01-23

**Authors:** SANG KYU LEE, YOUNG SANG KIM

**Affiliations:** Department of Biochemistry, College of Natural Sciences, Chungnam National University, Daejeon 305-764, Republic of Korea

**Keywords:** statins, endoplasmic reticulum stress, apoptosis, p53, eIF2α

## Abstract

Statins are effective cholesterol-lowering drugs that exert pleiotropic effects, including cytotoxicity to cancer cells. We previously reported that simvastatin triggered the mitochondrial apoptotic pathway in MethA fibrosarcoma cells, which was accompanied by the translocation of stabilized p53 to the mitochondria. In this study, we investigated whether statins induce the endoplasmic reticulum (ER) stress response and the mechanisms by which this response is linked to the stabilization of p53 and its translocation to the mitochondria. Statins induced typical ER stress-related proteins, such as BiP/78 kDa glucose-regulated protein (Grp78) and CCAAT/ enhancer-binding protein homologous protein (CHOP), as well as the phosphorylation of protein kinase RNA-like endoplasmic reticulum kinase (PERK), eIF2α and JNK. The statin-induced phosphorylation of eIF2α and JNK was inhibited by supplementation with components of the mevalonate pathway, such as mevalonate, farnesyl pyrophosphate (FPP) and geranylgeranyl pyrophosphate (GGPP). Salubrinal, an inhibitor of the dephosphorylation of eIF2α, suppressed the loss of mitochondrial membrane potential and the translocation of stabilized p53 and Bax to the mitochondria; however, SP600125, a JNK kinase inhibitor, did not exert this effect. Furthermore, the eIF2α knockdown sensitized cells to simvastatin-induced apoptosis and the overexpression of a non-phosphorylatable eIF2α-mutant [serine 51(Ser51)/alanine] enhanced the stabilization of p53 and its translocation to the mitochondria in response to simvastatin treatment. Taken together, these data indicate that eIF2α phosphorylation in the context of the ER stress response plays a role in cell survival by counteracting the p53-mediated mitochondrial apoptosis in response to statins.

## Introduction

Statins inhibit β-hydroxy-β-methylglutaryl CoA (HMG-CoA) reductase, which converts HMG-CoA to mevalonate. They are effective cholesterol-lowering drugs and exhibit anti-cancer effects by inducing apoptosis and cell cycle arrest (1). Moreover, the inhibition of the mevalonate pathway by statins causes perturbation of the endoplasmic reticulum (ER) and stress. In response to ER dysfunction, cells combat the stress and restore ER homeostasis by means of the unfolded protein response (UPR), which includes ER-associated degradation and control of translation (2,3). Among various ER responses, eIF2α phosphorylation primarily protects cells from stress by attenuating global translation and specifically upregulating chaperone proteins, although under prolonged and severe stress it leads to apoptosis (4). Statin-induced eIF2α phosphorylation has been shown to protect macrophages from hypoxia-induced cell death (5); however, lovastatin-induced eIF2α phosphorylation has been shown to lead to apoptosis in human head and neck squamous cell carcinoma (6). Elucidating the role of eIF2α phosphorylation induced by statins may lead to the development of novel protective and therapeutic approaches against hypercholesterolemia and cancer.

Under various stress conditions, the tumor suppressor p53 plays a pivotal role in the execution of ER stress-induced apoptosis via the activation of the BH3-only proteins, such as Puma and Noxa, in a transcription-dependent manner (7) and via a transcription-independent pathway; it activates members of the pro-apoptotic Bcl-2 family, such as Bax, Bid and Bak, or their translocation to the mitochondrial membrane (8). In our previous study, we demonstrated that simvastatin induced apoptosis in cancer cells by stabilizing p53 and stimulating its translocation with Bax to the mitochondria, resulting in the release of cytochrome *c*(9). However, the mechanisms by which the ER stress response, particularly eIF2α phosphorylation, is linked to the p53-mediated mitochondrial apoptotic pathway in statin-induced apoptosis, have not been investigated.

In the present study, we investigated the molecular link between eIF2α phosphorylation in the ER stress response and the p53 transcription-independent mitochondrial apoptotic pathway in the statin-induced apoptosis of MethA fibrosarcoma cells. We report that the eIF2α phosphorylation on serine 51 (Ser51) of the ER stress response attenuates cell death by inhibiting the stabilization of p53 and its translocation to the mitochondria in statin-induced apoptosis.

## Materials and methods

### Cells and reagents

Mouse MethA fibrosarcoma cells were maintained in RPMI-1640 (Invitrogen, Carlsbad, CA, USA) supplemented with 5% fetal bovine serum, 100 U/ml penicillin and 10 *μ*g/ml streptomycin at 37°C and 5% CO_2_. Simvastatin and lovastatin (MSD Korea, Ansan, Korea) were reconstituted in absolute ethanol and stored at −20°C. Mevalonolactone, farnesyl pyrophosphate (FPP) and geranylgeranyl pyrophosphate (GGPP) were purchased from Sigma-Aldrich (St. Louis, MO, USA). Salubrinal, tumnicamycin and SP600125 were obtained from Calbiochem (San Diego, CA, USA) and anti-tubulin antibody (T5186) from Sigma-Aldrich. Antibodies against p53, Bax, protein kinase RNA-like endoplasmic reticulum kinase (PERK), phospho-PERK, eIF2α, phosphoeIF2α, CCAAT/enhancer-binding protein homologous protein (CHOP)/GADD153, BiP/78 kDa glucose-regulated protein (Grp78), HRP-conjugated goat anti-mouse antibody and HRP-conjugated goat anti-rabbit antibody were supplied by Santa Cruz Biotechnology (Santa Cruz, CA, USA), while antibodies against phospho-JNK, total JNK and heat-shock protein (HSP) 60 were obtained from BD Biosciences (San Diego, CA, USA).

### Cell fractionation and western blot analysis

Cell fractionation was performed with a Mitochondria Isolation kit (Pierce, Rockford, IL, USA) according to the manufacturer’s instructions. For western blot analysis, cells were harvested, washed with ice-cold PBS and lysed in RIPA buffer [10 mM Tris (pH 7.4), 150 mM NaCl, 0.5% NP-40, 0.1% deoxycholate, 1 mM PMSF, 2 mM sodium fluoride and 1 mM sodium orthovanadate] for 15 min. Samples (3–30 *μ*g) were then subjected to SDS-PAGE and western blot analysis was performed using primary antibodies and HRP-conjugated secondary antibodies, followed by detection with West-Pico Chemiluminescent Substrates (Pierce) in the dark.

### Knockdown experiments and site-directed mutagenesis

siRNAs directed against eIF2α and CHOP were purchased from Santa Cruz Biotechnology and transiently transfected into MethA cells using Lipofectamine 2000 (Invitrogen). Stable clones were selected in the presence of 4 *μ*g/ml puromycin and screened by western blot analysis or RT-PCR. Substitution of the residue serine 51 of eIF2α with alanine (Ala) was performed using the QuikChange™ Site-Directed Mutagenesis kit (Stratagene, La Jolla, CA, USA). Briefly, wild-type eIF2α was amplified from MethA cDNA by PCR and cloned into the *Eco*RI and *Xho*I sites of pBluescript SK(+) vector (Stratagene). The mutant form of eIF2α was amplified from wild-type eIF2α/pBluescript SK(+) with Pfu polymerase (Intron, Seongnam, Korea) using the following mutagenesis primers and PCR conditions: forward, 5′-gcg aat tca tgc cgg ggc taa gtt gta g-3′; reverse, 5′-cgc tcg agt taa tct tca gct ttg gct t-3′; 18 cycles of 30 sec at 95°C, 60 sec at 55°C and 10 min at 68°C. The PCR product was *Dpn*I (Promega)-treated and transformed into DH5α competent cells and the substitution was confirmed by DNA sequencing. The wild-type and mutant forms of eIF2α were subcloned into the *Eco*RI and *Xho*I sites of the pCMV-tag2b vector (Stratagene) and transfected into the MethA cells. Stable clones of the eIF2α wild-type and mutant forms were selected in the presence of 4 mg/ml neomycin and screened by western blot analysis.

### Measurement of subgenomic content and mitochondrial membrane potential (MMP)

To analyze subgenomic content, cells were incubated under the indicated conditions, washed with PBS, fixed with 70% ice-cold ethanol at 4°C for 1 h and stained with 50 *μ*g/ml propidium iodide (PI) (Sigma-Aldrich) containing 100 *μ*g/ml RNase (Sigma-Aldrich) at 37°C for 30 min. DNA content was analyzed by a FACSCalibur (Becton-Dickinson, San Jose, CA, USA). To analyze MMP, cells were washed with PBS and stained with 500 *μ*l of 4 *μ*g/ml rhodamine-123 solution at 37°C for 20 min. They were then incubated with 0.1 mg/ml PI stock solution for 3 min. Incorporation of rhodamine-123 and PI was analyzed by a FACSCalibur. The green fluorescence of rhodamine-123 and the red fluorescence of PI were collected over the range of FL1 and FL2, respectively. All data were calculated using CellQuest software (Becton-Dickinson).

### Immunofluorescence

MethA cells were plated (1×10^5^ cells) on poly-L-lysine-coated coverslips (BD Biosciences) and treated with the indicated reagents for 12 or 36 h. Following incubation, the cells were washed with PBS, stained with MitoTracker Red (Molecular Probes, Eugene, OR, USA) for 20 min, washed twice with PBS, fixed, and permeabilized with Cytofix/Cytoperm solution (BD Biosciences) for 20 min at 4°C. The cells were washed with PBS and blocked with PBS containing 1% BSA for 20 min. They were then incubated with primary antibodies (anti-p53 and anti-Bax) diluted in 1% BSA/PBS for 1 h at RT. After washing with PBS 5 times, cells were incubated with anti-rabbit conjugated Alexa 488 (Molecular Probes) for 1 h at RT and stained with 1 *μ*g/ml Hoechst dye (Molecular Probes) for 10 min. After washing with PBS, the coverslips were mounted using Vectashield HardSet (Vector Laboratories, Burlingame, CA, USA) on glass slides and analyzed under a laser scanning microscope (LSM 5; Carl Zeiss, Oberkochen, Germany).

## Results

### Statins induce apoptosis and ER stress response by depletion of the isoprenyl products of the mevalonate pathway

**Figure 1:**
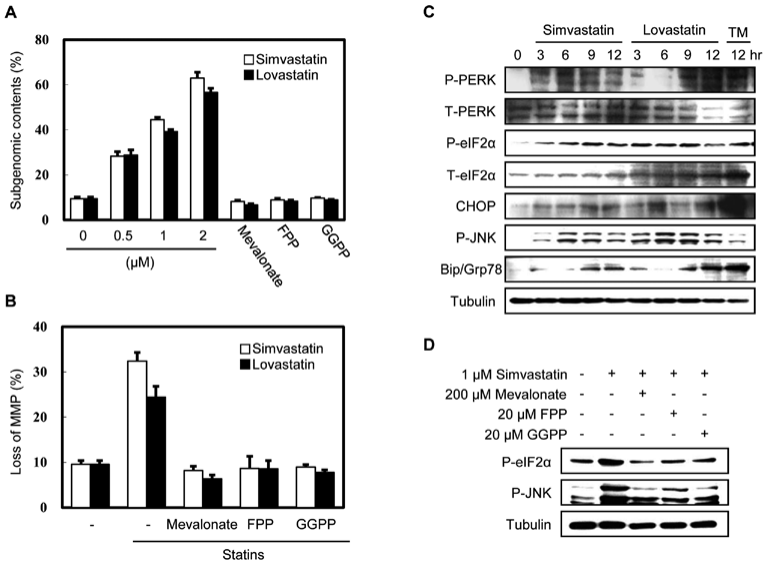
Statins induce apoptosis and ER stress responses by inhibiting the mevalonate pathway. (A) MethA cells were exposed to 0.5–2 *μ*M simvastatin or lovastatin for 24 h or treated with mevalonate (200 *μ*M), FPP (20 *μ*M) and GGPP (20 *μ*M) with 1 *μ*M simvastatin or lovastatin for 24 h and DNA content was analyzed by flow cytometry following PI staining. (B) MethA cells were incubated under the indicated conditions for 16 h and relative fluorescence intensity was analyzed by flow cytometry following staining with rhodamine-123 to evaluate the loss of MMP. (C) MethA cells were treated with 1 *μ*M lovastatin or simvastatin for the indicated times. Whole-cell extracts were analyzed for phosphorylation of PERK and eIF2α, expression of CHOP and Bip/Grp78 by western blot analysis using antibodies against phospho-PERK, total PERK, phospho-eIF2α, total eIF2α, CHOP, phospho-JNK, BiP/Grp78 and tubulin. TM; 2 nM tunicamycin. (D) MethA cells were treated with the indicated reagents for 9 h and assessed by western blot analysis with antibodies specific for phosphoeIF2α, phospho-JNK and tubulin.

To evaluate the effect of statins on subgenomic content and MMP, we treated MethA fibrosarcoma cells with simvastatin (a natural statin) or lovastatin (a synthetic statin) for 24 h and analyzed DNA fragmentation and changes in MMP. As shown in Fig. 1, both simvastatin and lovastatin induced DNA fragmentation in a dose-dependent manner and disrupted MMP, while these effects were completely inhibited by supplementation with downstream products of the mevalonate pathway, such as mevalonate, FPP and GGPP. Supplementation with squalene, the direct precursor of cholesterol, was ineffective, indicating that the level of cholesterol was not critical for apoptosis (data not shown), but that the key factor was the depletion of isoprenoids of the mevalonate pathway. To investigate whether the statin-induced apoptosis was related to ER stress, we examined several cardinal indicators of ER stress in statin-treated MethA cells by immunoblot analysis (Fig. 1C). We discovered that the CHOP and BiP/Grp78 protein expression was induced and that the phosphorylation of PERK, eIF2α and JNK was increased. These results indicate that statins induce a general ER stress response during apoptosis.

To determine which downstream products of the mevalonate pathway are critical for statin-induced ER stress, we examined the effects of mevalonate, FPP and GGPP supplementation on simvastatin-treated MethA cells. As shown in Fig. 1D, these downstream products of the mevalonate pathway reduced the phosphorylation of eIF2α and JNK, suggesting that the depletion of isoprenoids induced by statins disrupts ER homeostasis.

### Inhibition of eIF2α dephosphorylation opposes statin-induced apoptosis

**Figure 2:**
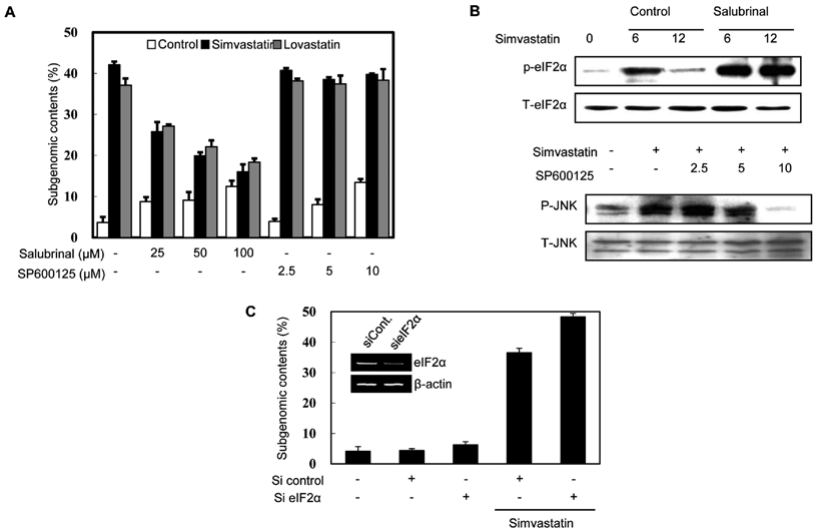
Inhibition of dephosphorylation of eIF2α reduces statin-induced apoptosis. (A) MethA cells were pre-incubated with various concentrations of salubrinal (25–100 *μ*M), a selective inhibitor of eIF2α dephosphorylation, or SP600125 (2.5–10 *μ*M), a specific inhibitor of JNK kinase, for 30 min and were then treated with 1 *μ*M simvastatin or lovastatin for 24 h. Following incubation, the cells were stained with PI and subgenomic DNA content was assessed by flow cytometry. (B) MethA cells were pre-incubated with 50 *μ*M salubrinal for 30 min and then treated with 1 *μ*M simvastatin for the indicated times. Phosphorylation of eIF2α was detected by western blot analysis using antibodies against phospho-eIF2α (p-eIF2α) and total-eIF2α (T-eIF2α). MethA cells were pre-incubated with 2.5–10 *μ*M SP600125 for 30 min and then treated with 1 *μ*M simvastatin for 6 h. Phospho-JNK and total JNK were assessed by western blot analysis. (C) MethA cells were transfected with control (siCont.) or eIF2α-siRNA (sieIF2α) for 48 h and then treated with 1 *μ*M simvastatin for 24 h. Following incubation, the cells were stained with PI and subgenomic DNA content was assessed by flow cytometry. Expression of eIF2α mRNA was also measured by RT-PCR (inset).

To investigate whether the phosphorylation of eIF2α and JNK induced by statins is involved in apoptosis, we assessed the effects of salubrinal (a selective inhibitor of eIF2α dephosphorylation) and SP600125 (a specific inhibitor of JNK kinase). Notably, salubrinal treatment increased the phosphorylation of eIF2α and reduced the DNA fragmentation induced by statins (Fig. 2A and B), whereas SP600125 treatment had no effect, although it clearly inhibited JNK phosphorylation (Fig. 2A and B). This result suggests that the phosphorylation of eIF2α, but not that of JNK, plays a role in statin-induced apoptosis. To confirm this finding, we investigated the effect of the eIF2α knockdown on DNA fragmentation. As shown Fig. 2C, the MethA cells in which eIF2α had been knocked down were more sensitive to simvastatin-induced apoptosis than the mock-transfected cells. Taken together, these results support the notion that the phosphorylation of eIF2α negatively modulates statin-induced apoptosis in MethA cells.

### Inhibition of eIF2α dephosphorylation decreases the stabilization of p53 and its translocation to the mitochondria in statin-induced apoptosis

**Figure 3:**
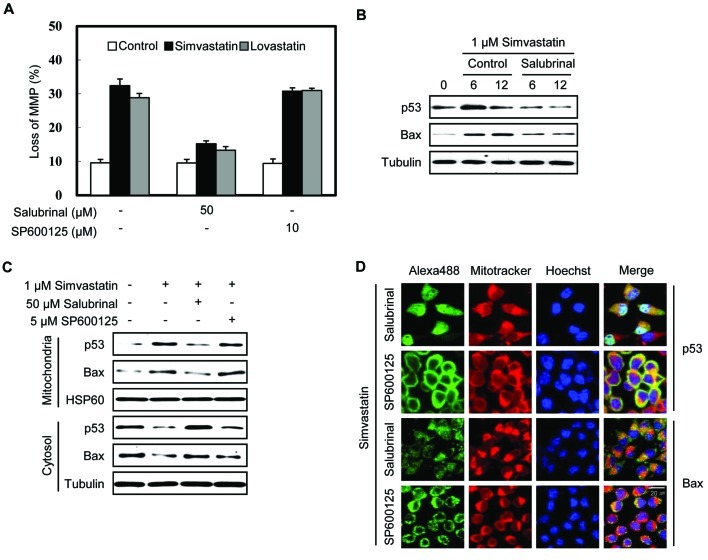
Inhibition of eIF2α dephosphorylation by salubrinal decreases the stabilization and translocation of p53 to the mitochondria in simvastatin-induced apoptosis. (A) MethA cells pre-incubated with 50 *μ*M salubrinal or 10 *μ*M SP600125 for 30 min were treated with 1 *μ*M simvastatin or lovastatin for 16 h. The cells were then stained with rhodamine-123 and relative fluorescence intensity was analyzed by flow cytometry to measure the loss of MMP. (B) MethA cells were pre-incubated with 50 *μ*M salubrinal for 30 min and were then treated with 1 *μ*M simvastatin for the indicated time periods. The level of p53 and Bax proteins were assessed by western blot analysis. (C) MethA cells were pre-incubated with 50 *μ*M salubrinal or 5 *μ*M SP600125 for 30 min and then treated with 1 *μ*M simvastatin for 12 h. The cells were divided into cytosolic and mitochondrial fractions and the levels of p53 and Bax in the cytosolic and mitochondrial fractions were assessed by western blot analysis. (D) MethA cells were incubated under the indicated conditions for 12 h and immunostained with anti-p53 and anti-Bax antibody (green). p53 and Bax were observed under a confocal microscope at ×630 magnification. Nuclear staining with Hoechst dye (blue) and mitochondrial staining with MitoTracker (red) are shown.

We previously reported that simvastatin activates the mitochondrial apoptotic pathway in MethA cells accompanied by the stabilization of the p53 protein and its translocation with Bax to the mitochondria and that the knockdown of p53 expression decreases apoptosis (9). To determine whether the phosphorylation of eIF2α and JNK contributes to the simvastatin-induced MMP loss and translocation of p53 to the mitochondria, we treated the cells with salubrinal (an inhibitor of the dephosphorylation of eIF2α) and SP600125 (a JNK kinase inhibitor). Salubrinal treatment significantly reduced statin-induced MMP loss (Fig. 3A) and the stabilization of p53 and Bax by simvastatin in the MethA cells (Fig. 3B), as well as the simvastatin-induced translocation of p53 and Bax to the mitochondria (Fig. 3C). By contrast, SP600125 had no discernible effect. The inhibitory effect of salubrinal on the simvastatin-induced translocation of p53 and Bax to the mitochondria was also evident via immunofluorescence staining (Fig. 3D). In the presence of salubrinal, the p53 and Bax mitochondrial co-localization was less apparent than in the presence of SP600125. These results demonstrate that the phosphorylation of eIF2α counteracts the p53-mediated mitochondrial apoptotic pathway in simvastatin-induced apoptosis.

### Phosphorylation of eIF2α on Ser51 is responsible for the stabilization of p53 and its translocation to the mitochondria

**Figure 4:**
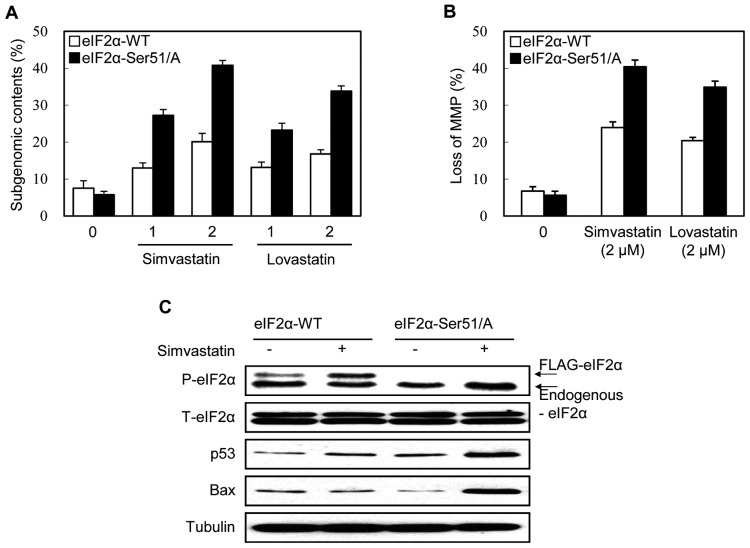
Phosphorylation of eIF2α on serine 51 (Ser51) decreases the stabilization and translocation of p53 to the mitochondria in statin-induced apoptosis. MethA cells transfected with wild-type eIF2α (eIF2α-WT) and cells transfected with the eIF2α mutant (Ser51/A) were exposed to 1–2 *μ*M simvastatin or lovastatin for 24 h. (A) Subgenomic DNA content and (B) loss of MMP were analyzed by flow cytometry. (C) MethA cells transfected with wild-type or mutant eIF2α were treated with 1 *μ*M simvastatin for 9 h. Phosphorylation of eIF2α and expression of p53 and Bax were examined by western blot analysis.

Several kinases, including PERK, phosphorylate eIF2α on Ser51 in response to various ER stress inducers. To investigate whether the phosphorylation of eIF2α on Ser51 is involved in the statin-induced ER stress response and apoptosis, we examined the effect of the overexpression of the non-phosphorylatable mutant form of eIF2α (Ser51/Ala) on simvastatin-induced apoptosis. The overexpression of the mutant eIF2α (Ser51/Ala) attenuates the eIF2α phosphorylation pathway (10). The flag-tagged wild-type eIF2α-transfected clone was used as the control. Both the DNA fragmentation and MMP loss induced by simvastatin were enhanced in the eIF2α mutant-expressing clone compared to the wild-type-expressing clone (Fig. 4A and B). The effect of the overexpression of the eIF2α mutant on p53 and Bax protein in response to simvastatin was also analyzed by immunoblot analysis. As shown in Fig. 4C, simvastatin treatment induced the phosphorylation of the flag-tagged eIF2α wild-type protein, but not that of the flag-tagged mutant protein. At the same time, simvastatin effectively stabilized p53 and Bax proteins in the flag-tagged eIF2α mutant clone compared with the flag-tagged eIF2α wild-type clone (Fig. 4C). These results indicate that the phosphorylation of eIF2α on Ser51 is responsible for the cell survival effect in the p53-mediated mitochondrial apoptosis of statin-treated MethA cells.

## Discussion

Under stress conditions, p53 is stabilized and acts as a transcription factor for the expression of pro-apoptotic target genes, such as Puma, Noxa, Bax and Bid (11). In addition, cytoplasmic p53 directly activates the mitochondrial apoptotic pathway in a transcription-independent manner; i.e., p53 interacts with the Bcl-2 family members, Bcl-2 or Bcl-xL, leading to the translocation of Bax and Bid to the mitochondrial outer membrane (12). We previously reported that in response to statin, p53 itself is stabilized and translocated along with Bax to the mitochondria, thus activating the mitochondrial apoptotic pathway (9). In the present study, we demonstrate that statins induce the ER stress response in MethA cells by depleting the isoprenyl products of the mevalonate pathway and that the phosphorylation of eIF2α on Ser51 plays a role in promoting cell survival. To our knowledge, this is the first study to demonstrate that the phosphorylation of eIF2α counteracts the stabilization of p53 and its translocation to the mitochondria in statin-induced apoptosis.

Statins exert stress on cancer cells and display various cardinal features of ER stress response, which are pro- or anti-apoptotic. Lovastatin has been shown to induce apoptosis accompanied by reduced global protein translation by inducing eIF2α phosphorylation. It has also been shown to induce general control non-repressed 2 (GCN2)-mediated activating transcription factor (ATF)4 production followed by the increased expression of ATF3 and CHOP in a head and neck squamous cell carcinomas cell line (6). Lovastatin-induced apoptosis was attenuated in CHOP^−/−^ and GCN2^−/−^ murine embryonic fibroblasts (MEFs), thus demonstrating the involvement of ATF4, CHOP and ATF3 in apoptosis. In multiple myeloma cells, lovastatin has been shown to induce BiP/Grp78 and CHOP protein expression, the phosphorylation of eIF2α and apoptosis, as well as the cleavage of poly(ADP-ribose) polymerase (PARP) (13). In contrast to these apoptotic effects of statin-induced ER stress, numerous studies have demonstrated that the ER stress response also attenuates the apoptotic response. Fluvastatin has been shown to induce BiP/Grp78 expression and to activate ATF6 and X-box binding protein-1 (XBP-1), but not CHOP and ATF4 in RAW264.7 cells (5). Among the induced proteins, the induction of BiP/ Grp78 is responsible for the cytoprotective effect of fluvastatin pre-treatment against hypoxia-induced cell death. Simvastatin pre-treatment also exerted a neuroprotective effect by attenuating the ER stress response, with concomitant increases in ATF6 and XBP-1 protein expression during acute ischemia and reperfusion in rats (14). Recently, a novel protective effect of statins against atherosclerosis was proposed based on the finding that stearic acid-induced ER stress in macrophages was attenuated by statins (15). In sum, statins seem to exert differential ER stress responses depending on the strength, nature and duration of the stress. However, we observed that statins induce the majority of indicators of the ER stress response in MethA cells: the early phosphorylation of PERK, eIF2α and JNK and the induction of CHOP and BiP/Grp78 protein expression.

The induction of CHOP and JNK phosphorylation suggests that, under our experimental conditions, ER stress in response to statins triggers apoptotic signals. The MethA clone in which CHOP was knocked down by siRNA exhibited increased resistance to statin-induced apoptosis, pointing to a pro-apoptotic role of CHOP (data not shown). However, the JNK inhibitor, SP600125 did not exert any effect on statin-induced apoptosis in our study (Figs. 2 and 3). The JNK kinase pathway is activated during lovastatin-induced apoptosis in the NB4 acute promyelocytic leukemia cell line (16) and human breast cancer cells (17). By contrast, the JNK pathway is downregulated (18) or not affected (19) in statin-induced apoptosis. Further study is required to elucidate the role of JNK phosphorylation in statin-induced ER stress.

Salubrinal, an inhibitor of eIF2α dephosphorylation, clearly attenuated statin-induced apoptosis, suggesting that the phosphorylation of eIF2α plays a role in triggering cell survival signals. Previous studies have also demonstrated that phosphorylation of eIF2α protected cells under glucose deprivation stress (20,21), and other ER stress (22). When global translation is inhibited by the phosphorylation of eIF2α, the translation of certain mRNAs, such as GCN2, ATF4 and X-linked inhibitor of apoptosis protein (XIAP), is increased and this promotes tumor cell survival and chemoresistance (23,24). In renal medullary cells exposed to urea stress, the phosphorylation of eIF2α by activated GCN2 also exerts cytoprotective effects (25). On the other hand, there have been several reports demonstrating that the phosphorylation of eIF2α is involved in apoptosis under various stress conditions, such as proteasome inhibition (26,27), hypoxia (28) and tunicamycin (10). Moreover, salubrinal enhances cisplatin-induced nephrotoxicity (29). Depending on the nature and strength of the stress, the phosphorylation of eIF2α seems to shift from its primary role of cytoprotection to apoptosis induction (4). More importantly, further studies are required to elucidate the signaling flows from the phosphorylation of eIF2α to the reduced stability of p53 in response to statins. Unveiling the missing link between p53 stabilization and eIF2α phosphorylation may contribute to the expansion of therapeutic approaches against hypercholesterolemia and cancer.
